# Changes in taste perception in elderly population and its potential impact on oral health: a systematic review with meta-analysis

**DOI:** 10.3389/froh.2024.1517913

**Published:** 2024-12-05

**Authors:** Larisse Santos Mendonça Alves, Júlia Maria de Sousa Munduri, Mariana Caldas de Oliveira Mattos, Cristine Miron Stefani, Naile Dame-Teixeira

**Affiliations:** Department of Dentistry, School of Health Sciences, University of Brasilia, Brasilia, Brazil

**Keywords:** aging, taste disorders, altered taste, oral health, saliva, systematic review

## Abstract

**Aim:**

Gustatory function plays a fundamental role in various aspects related to nutrition and health, and the decline in taste perception can result in a series of adverse consequences. This is expected with aging due to a decrease in taste buds and other conditions, leading to systemic and oral diseases. We aimed to compare taste sensitivity in the elderly population vs. adults.

**Methods:**

This systematic review was reported according to PRISMA guidelines. The search was performed in four databases, as well as in grey literature. The risk of bias was assessed using the JBI's critical appraisal tools for observational studies. A meta-analysis with subgroups according to each flavor was conducted to obtain differences in means for adults vs. elderly (random-effects model).

**Results:**

Out of the 5,660 studies retrieved, 18 observational studies were included, representing a total of 1,680 aged 18–59 years and 1,048 aged ≥ 60 years. Elderly individuals need higher concentrations to distinguish flavors compared to adults. In a qualitative analysis, all flavors showed differences between the groups, with sweet flavor being the easiest to recognize and the thresholds between the groups not being highly discrepant. However, in the meta-analysis, statistical differences were observed for sweet, salty, and umami flavors, while there were no statistical differences for sour and bitter flavors between the groups.

**Conclusions:**

There are significant differences for distinguishing sweet, salty, and umami flavors between adults and elderly. Bitter and sour flavors did not exhibit differences in elderly.

**Systematic Review Registration:**

https://www.crd.york.ac.uk/prospero/display_record.php?RecordID=463873, PROSPERO (CRD42023463873).

## 1 Introduction

Advancements in technology and science have led to a steady increase in life expectancy, making healthy aging essential for maintaining quality of life. According to the World Health Organization (WHO), the global population aged 60 years and older surpassed one billion in 2019, accounting for 13.2% of the total world population. This figure represents a 2.5-fold increase since 1980, when there were 382 million individuals aged 60 and above. Projections indicate that by 2050, this number could reach nearly 2.1 billion worldwide (1). One notable aspect of aging is the decline in taste perception, which can negatively affect the dietary habits of older adults (2). The human gustatory system can recognize five basic tastes: sweet, sour, bitter, salty, and umami (3), and this taste perception is vital for various aspects of nutrition and health. Decline in teste perception can lead to numerous adverse outcomes including reduced appetite, lower food intake, altered dietary preferences, and an increased risk of malnutrition. Such changes can contribute to the development of serious health conditions, including hypertension, diabetes mellitus, and dyslipidemia, among others (4).

The most common causes of age-related declines in gustatory function include physiological changes such as reduced taste bud density, diminished function of taste receptor cells, challenges in maintaining oral health, decreased olfactory function, chronic diseases, and polypharmacy (5, 6). Oral causes that are associated with changes in people's diets and overall health include reduced salivary flow and tooth loss, the latter being caused by advanced caries lesions and/or periodontal disease (7). A high intake of sugar-added foods increases the risk of developing carious lesions, which can lead to tooth loss, impairing chewing ability and further altering dietary habits - creating a vicious cycle. For example, root caries is particularly prevalent among the elderly, elevating the likelihood of carious lesion development and tooth loss (8).

Physiological aging is also associated with the degeneration of functional tissue in salivary glands, resulting in a decrease in saliva production (9, 10). Saliva plays a fundamental role in oral functions, including both functional processes such as swallowing and speech, and the sensory perception of taste, acting in the preparation and transmission of gustatory stimuli (9, 11). Saliva characteristics vary widely among individuals throughout life, which may explain some of the differences in taste perception. For example, studies show that the response to sucrose and the perception of sweetness depend on saliva pH (10, 11).

Previous studies have shown that recognition thresholds for the five basic tastes are significantly higher in elderly individuals than in younger adults (2). Methven et al. (2012) conducted a systematic review with meta-analysis to investigate the worsening of taste perception in healthy aging and discuss the extent of change (12). However, this study addressed only briefly the relationship between oral health issues and sensory perception, and it is in need of an update. A more focused review on oral health is essential to understand how changes in macronutrient consumption directly impact oral health and how the dentistry clinicians can help break the previously mentioned vicious cycle. Additionally, assessing existing oral health problems is crucial for determining their association with changes in taste perception.

Addressing and managing the decline in taste perception among older adults can be essential for promoting healthy eating habits and enhancing their quality of life. In this systematic review, we aimed to compare taste sensitivity in the elderly population vs. adults. Given the potential impact on increased sugar consumption in sweetness perception changes and its relevance to dentistry, we excluded studies that did not evaluated sweet taste.

## 2 Methods

This systematic review protocol was registered at the PROSPERO International Prospective Register of Systematic Reviews platform under the number CRD42023463873. The acronym PECOS was used to design the following research question: “Do older adults experience alterations in taste perception when compared to adults?” where *P* = general population, *E* = aged people, *C* = adults, *O* = altered taste sensation, and *S* = observational and clinical studies.

### 2.1 Data source and search strategy

The following databases were searched: PubMed/MEDLINE, Embase, Web of Science, and Scopus. Also, the grey literature (ProQuest for dissertations and theses, Google Scholar, Livivo), and references lists of the included studies were searched. To assess alterations in taste perception in older adults compared to adults, the main terms added in the search strategy were “Old Age, Older adults, Aged”, “Taste Disorders, Dysgeusia, Tasting, Distorted Taste, Altered Taste, Taste Perception, Gustatory Perception, Gustatory Response, sweet taste, diet, carbohydrate restricted”. MESH terms were also included. The complete search strategy can be found in Supplementary Appendix 1.

### 2.2 Inclusion criteria

Observational and clinical studies, without limitation of publication date and language, assessing taste perception in at least one group of elderly individuals (≥60 years) compared to adults, using quantitative, qualitative, or hedonic scales as evaluation criteria, were included.

### 2.3 Exclusion criteria

Studies were excluded if: (1) the population included children and adolescents, edentulous individuals, participants with cancer and/or those who have undergone chemotherapy and radiation therapy, individuals with dementia or other neurological conditions, and studies that did not exclude participants continuously using medications that may alter taste perception. (2) Articles with unavailable full text, preclinical studies (*in vitro* and animal studies), case reports or case series studies, randomized studies, systematic reviews and other reviews (narrative, scoping), letters, editorials, opinions, books and book chapters, and conference abstracts. (3) Studies without a control group (adults) or test group (elderly person). (4) Studies that did not evaluate the sweet taste. (5) Studies that have not assessed taste perception using a quantitative/qualitative scale.

### 2.4 Studies selection

Two independent reviewers (LSMA and JMSM) selected the titles and abstracts for each study based on the eligibility criteria. Afterward, the same reviewers independently assessed the full text, confirming their eligibility. The discrepancies were resolved with the involvement of a third reviewer (MCOM). The Rayyan QCRI tool (Qatar Computer Research Institute, Qatar) was used in both phases of study selection.

### 2.5 Data extraction

Data extraction was performed by one reviewer (LSMA) and cross-checked by a second one (JMSM). Conflicts were solved by a third reviewer (MCOM). Extracted data included the first author, year of publication, the sample size of tests and controls, proportions of male and female participants, mean age of the participants in each group, methods of taste evaluation (quantitative scale, qualitative scale, hedonic scale), unit of measurement for this evaluation (Likert scale, minimal concentration to have taste perception, or threshold of recognition and detection), outcomes and findings for adults and elderly, main conclusions, and study type.

### 2.6 Qualitative data synthesis

Studies and their results were grouped and classified according to the type of unit of measurement (Likert scale, minimal concentration to have taste perception, and threshold of recognition and detection) to identify the flavors sweet, salty, sour, bitter, and umami, age of participants in the control/test group, sample size, number of participants in each group, proportion of female and male participants in each study, outcomes of each group, overall study conclusion, study type, and a field for observations to report missing data.

### 2.7 Quantitative data synthesis

A pair-wise meta-analysis was conducted, comparing the taste perception between aged people and adults, with subgroups according to basic tastes. The data analysis was conducted using the statistical program RevMan version 5.4.1 (13), and the statistical method used was the inverse variance with a random effects model. Due to substantial variability in methods in the included studies, the effect measured was the standardized mean difference with a 95% confidence interval. Heterogeneity among studies was estimated using Cochran's Q test, and inconsistency was assessed using the I^2^ statistic.

### 2.8 Risk assessment of individual studies

The same reviewers (LSMA and JMSM) assessed the methodological quality of individual studies using the JBI Critical Appraisal Checklist for Analytical Cross-Sectional Studies (14). All studies were assessed using the same tool as only one design was identified. This tool presents 8 items, of which 4 were considered critical domains for this systematic review, including “Were the study subjects and the setting described in detail?”; “Were objective, standard criteria used for measurement of the condition?”; “Were the criteria for inclusion in the sample clearly defined?”, and “Was the exposure measured in a valid and reliable way?”. The criteria adopted in this systematic review for considering an article as of high risk were answers of “no” or 1 “no” and 1 “unclear” or 2 “unclear” answers in critical domains, or 2 “unclear” answers and 1 or more “no” answers in non-critical domains. An article was considered to have low risk if it received at most 1 “no” answer or 2 “unclear” answers in non-critical domains. Decisions regarding critical and non-critical domains and classification systems were discussed and agreed upon with the research team before applying the tool, as described in the JBI Reviewer Manual.

## 3 Results

### 3.1 Study selection and qualitative synthesis

**Figure 1:**
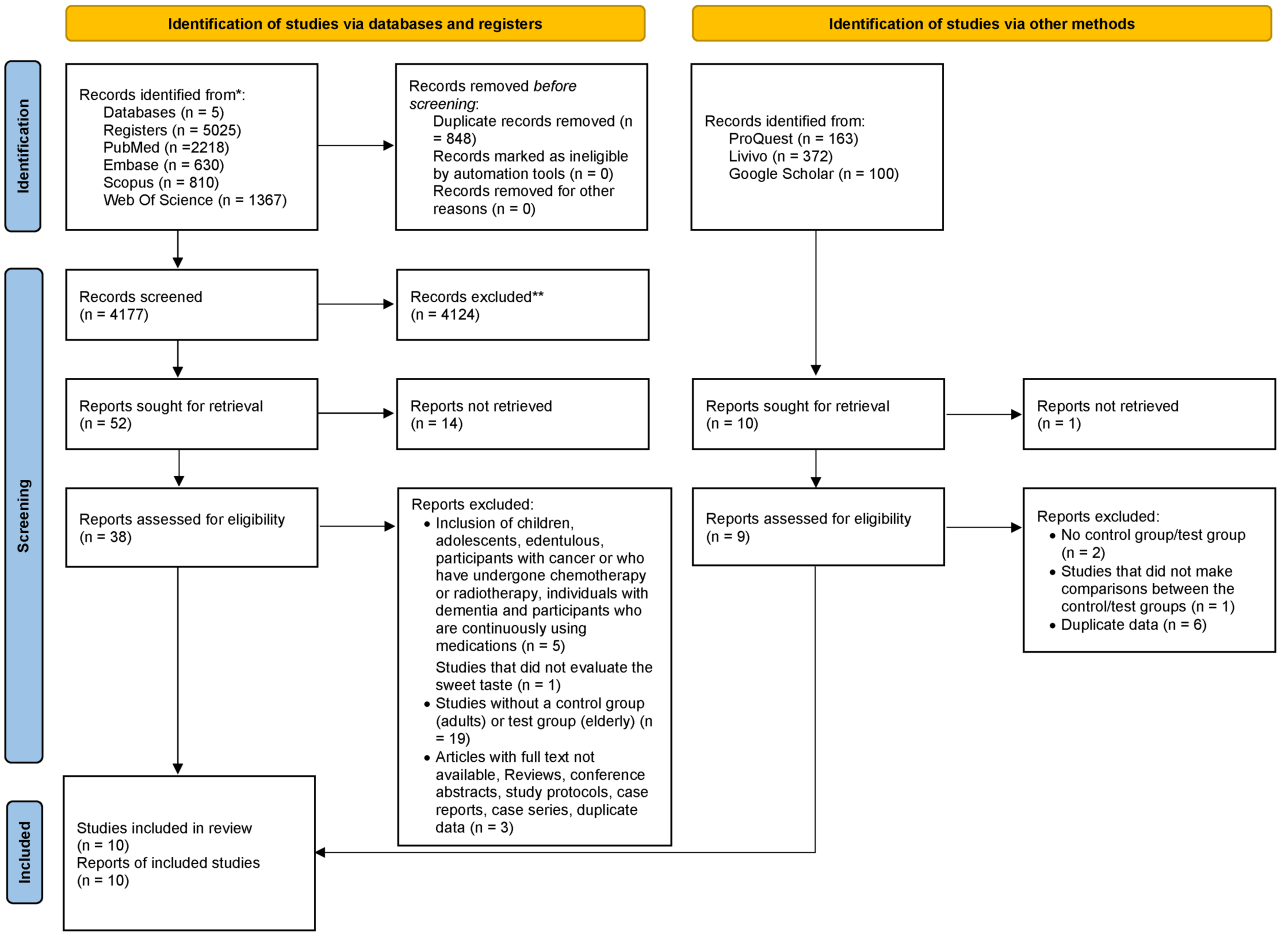
Flowchart of the study.

A total of 5,660 studies were identified through searching the main databases and grey literature. After excluding duplicates, 4,177 (main) and 545 (grey literature) studies were screened, of which 62 studies remained for full-text reading, and 18 were included in the final analysis (Figure 1). Supplementary Appendix 2 details the excluded studies with each reason for exclusion.

Table 1 shows the characteristics of the 18 included studies in a qualitative synthesis, including the number of participants in each group, the method used to assess taste perception, and the results. All studies were cross-sectional. A total of 2,728 individuals were included here, of which 1,680 were adults aged 18–59 years and 1,048 individuals aged ≥ 60 years. Similar numbers of female (*n* = 1,412) and male (*n* = 1,202) were reached. It was observed that 5 studies used Likert scales for taste sensation, 1 evaluated the minimum concentration for taste perception, and 15 assessed recognition/detection thresholds. The included studies were published from 1986 to 2022. A geographic analysis showed that most studies were conducted in the United States (6 studies reported in the USA, 3 in the Netherlands, 2 in Japan, 1 in England, 1 in Thailand, 1 in Spain, 1 in China, and 1 in South Korea).

**Table 1:** Characteristics of the included studies (*N* = 18).

Author; year	Country	Ref.	Risk assessment	*N* (C)	*N* (T)	Age (C)	Age (T)	sex	Taste measurement	Unit of measurement	Flavor	Outcome (C)	Outcome (T)
Bales et al. 1986	United States	(15)	+	30	32	18–30	≥60	Fem 69,4% Men 30,6%	Qualitative Scale	Detection threshold	Sweet and salty	Sweet (mM)- 7.2 ± 0.9	Sweet (mM) 10.2 ± 0.9
												Salty (mM)- 1.3 ± 0.2	Salty (mM) 4.9 ± 0.9
Weiffenbach et al. 1986	United States	(16)	+	85	85	<40–56	57–>70	Fem 46,5% Men 53,5%	Qualitative Scale	Detection threshold	Sweet, salty, sour and bitter	<40 years: Sweet 1.06 ± .38 Salty 1.42 ± .50 Sour 1.26 ± .50 Bitter 1.02 ± .47 40–56 years: Sweet 1.05 ± .39 Salty 1.20 ± .41 Sour 0.99 ± .46 Bitter 0.99 ± .51	57–70 years: Sweet 1.22 ± .46 Salty 1.12 ± .46 Sour 1.11 ± .42 Bitter 0.81 ± .36. > 70 years: Sweet 1.12 ± .54 Salty 1.12 ± .41 Sour 1.01 ± .41 Bitter 0.71 ± .41
Spitzer 1988	United States	(17)	+	15	17	18–25	63–88	Men 100%	Qualitative Scale	Detection threshold	Sweet, salty, sour and bitter	Sweet 8.74 ± 0.21 Salty 2.59 ± 0.36 Sour 0.08 ± 0.29 Bitter 2.64 ± 0.45	Sweet 9.07 ± 0.24 Salty 5.49 ± 0.33 Sour 0.13 ± 0.21 Bitter 10.46 ± 0.57
Cowart 1989	United States	(18)	+	58	29	19–60	65–80	Fem 55,2% Men 44,8%	Qualitative Scale	Likert scale	Sweet, salty, sour and bitter	Sweet: very intense at high concentration Salty: extremely intense at high concentration Sour: moderate at low concentration Bitter: Very strong at high concentration for middle-aged individuals and extremely strong at high concentration for young individuals	Sweet: very intense at high concentration Salty: very intense at high concentration Sour: slightly strong at low concentration Bitter: very intense at high concentration
Gilmore et al. 1989	United States	(19)	+	12	12	18–25	67–77	Fem 100%	Qualitative Scale	Minimum concentration	Sweet, and bitter	Sweet e Bitter: identified at low concentrations	Sweet: identified at low concentrations Bitter: Identified at medium to high concentrations
Stevens 1995	United States	(20)	+	15	15	19–26	65–87	–	Qualitative Scale	Detection threshold	Sweet	Sweet: Detection at a concentration of 55:1 (dilution 55 times less than the most concentrated solution)	Sweet: Detected at a concentration of 100:1 (corresponding to a concentration range 100 times higher than the most concentrated solution)
Receputo et al. 1996	Italy	(21)	+	20	40	27.7 + 3.2	Idosos -71,3 + 5,5 Centenários- 102.6 + 2.4	Fem 60% Men 40%	Qualitative Scala	Detection threshold	Sweet, salty, sour and bitter	Ability to identify the flavors Sweet: 97.5% Salty: 92,5% Sour: 92,5% Bitter: 87,5%	Ability to identify the flavors Elderly Sweet: 69% Salty: 68% Sour: 77% Bitter: 78% Centenarians Sweet: 46% Salty: 49% Sour: 56% Bitter: 63%
Keneda et al. 2000	Japan	(22)	+	20	20	21–40	59–75	Fem 50% Men 50%	Qualititative Scale	Detection threshold	Sweet and sour	Sweet: higher thresholds Sour: lower thresholds Concentrations (sweet and sour)	Sweet: higher thresholds Sour: higher thresholds Concentrations (sweet and sour)
												High: High percentage of correct identifications Moderate: High percentage of correct identifications Low: Low percentage of correct identifications	High: High percentage of correct identifications Moderate: Significantly lower percentage of correct identifications Low: Low percentage of correct identifications
Mojet et al. 2001	Netherlands	(23)	+++	21	21	19–33	60–75	Fem 50% Men 50%	Qualitative Scale	Detection threshold	Sweet, salty, sour, bitter and umami	Sweet, salty, sour, bitter e umami: Significantly lower detection thresholds	Sweet, salty, sour, bitter e umami: Significantly higher detection thresholds
Mojet et al. 2003	Netherlands	(24)	+++	21	21	19–33	60–75	Fem 50% Men 50%	Qualitative Scale	Detection threshold	Sweet, salty, sour, bitter and umami	Sweet, salty, sour, bitter and umami: they perceived the flavors as significantly more intense when dissolved in water and in the product	Sweet, salty, sour, bitter and umami: they perceived the flavors as significantly less intense when dissolved in water and in the product
Fukunaga et al. 2005	Japan	(25)	+	30	30	18–29	65–85	Fem 56,7% Men 43,3%	Qualitative Scale	Detection threshold	Sweet, salty, sour and bitter	Sweet, salty, sour and bitter: Lower threshold	Sweet, salty, sour and bitter: Higher threshold
Mojet et al. 2005	Netherlands	(26)	+++	21	21	19–33	60–75	Fem 50% Men 50%	Qualitative Scale	Detection threshold; Likert scale	Sweet, salty, sour, bitter and umami	Compounds:	Compounds:
												Water	Water
												Sweet Sucrose 4.61 ± 0.60 Aspartame 4.64 ± 0.53	Sweet Sucrose 4.04 ± 0.86 Aspartame 4.21 ± 1.03
												Salty: Sodium chloride 6.22 ± 0.81 Sour: Citric acid 6.08 ± 0.84	Salty: Sodium chloride 5.27 ± 0.72 Sour: Citric acid 5.50 ± 1.01
												Bitter: Caffeine 3.45 ± 0.70 Umami: Monosodium glutamate 3.93 ± 0.85 Products Sweet	Bitter: Caffeine 2.77 ± 1.09 Umami: Monosodium glutamate 2.79 ± 0.76 Product Sweet
												Sucrose 6.24 ± 0.74	Sucrose 5.36 ± 1.18
												Aspartame 4.46 ± 0.69	Aspartame 3.80 ± 1.11
												Salty: Sodium chloride 14 ± 0.94	Salty: Sodium chloride 4.12 ± 0.97
												Sour: Citric acid 4.62 ± 1.06	Sour: Citric acid 4.06 ± 1.27
												Bitter: Caffeine 4.08 ± 1.24	Bitter: Caffeine 4.40 ± 1.47
												Umami: Monosodium glutamate 4.84 ± 1.29	Umami: Monosodium glutamate 4.34 ± 1.19
Kennedy et al. 2010	England	(27)	+	36	48	18–33	63–85	–	Quantitative Scale/ Hedonic Scale	Detection threshold/recognition; Hedonic scale; Likert scale	Sweet	Sweet: More sensitive to taste; Hedonic test: chocolate more liked than the sucrose solution; Intensity: higher sweetness intensities of sucrose	Sweet: Less sensitive to taste; Hedonic test: chocolate more liked than the sucrose solution; Intensity: lower sweetness intensities of sucrose
Wiriyawattana et al. 2018	Thailand	(4)	+	60	30	20–59	60–85	Fem 70% Men 30%	Qualitative Scale;	Detection threshold/recognition	Sweet, salty, sour, bitter and umami	Sweet, salty, sour, bitter and umami: lower thresholds	Sweet, salty, sour, bitter and umami: higher thresholds
Barragán et al. 2018	Spain	(28)	+	671	349	18–50	51–80	Fem 64,2% Men 35,8%	Quantitative Scale	Likert scale	Sweet, salty, sour and bitter	Sweet, salty, sour and bitter: Perceived a stronger intensity with the same high concentration as the elderly	Sweet, salty, sour and bitter: Perceived weaker intensities at the same high concentration as the young Sour and Bitter: Lower flavor scores
Wang et al. 2020	Taiwan	(29)	+	160	80	20–59	≥60	Fem 50% Men 50%	Quantitative Scale; Quantitative Scale; Hedonic Scale	Detection threshold; Hedonic scale; Likert scale	Sweet, salty, sour and bitter	Sweet, salty, sour and bitter: Higher accuracy scores (age group 20 to 39 years); Intensity rating: higher scores (age range 20 to 39 years); Pleasantness: no differences between ages	Sweet, salty, sour and bitter: Lower accuracy scores; Intensity rating: lower scores; Pleasantness: no differences between ages
Jeon et al. 2021	South Korea	(2)	+	71	68	20–29	≥65	Men 100%	Qualitative Scale;	Recognition threshold	Sweet, salty, sour, bitter and umami	Sweet, salty, sour, bitter and umami: lower thresholds	Sweet: The same recognition threshold as adults. However, after analyzing the correlation with age, the threshold increased Salty, sour, bitter and umami: higher thresholds
Huang et al. 2022	China	(3)	+++	334	130	19–50	51–65	Fem 48,9% Men 51,1%	Hedonic Scale	Detection threshold/recognition; Likert scale	Sweet, salty, sour, bitter and umami	Sweet, salty, sour, bitter and umami: lower thresholds	Sweet, sour, bitter and umami: Significantly lower perception scores Sour, bitter and umami: Significantly lower recognition scores

C = Control (adults); T = Test (aged).

Risk assessment, as per JBI instrument: +++ = low risk of bias; ++ = moderate risk of bias; + = high risk of bias.

Regarding the flavors analyzed in the studies, 18 assessed sweet taste, 14 assessed salty, bitter, and sour tastes, and 7 assessed umami taste. The flavors were diluted in distilled water or in other products, with most studies using sucrose solutions for sweet taste, sodium chloride (NaCl) for salty taste, caffeine or quinine hydrochloride (HCl) for bitter taste, citric acid for sour taste, and monosodium glutamate (MSG) for umami taste. Since recruiting aged individuals without dental prostheses was challenging, some studies allowed those who used partial dentures, but they were instructed not to use them during the tests. Some studies also instructed participants to avoid eating and drinking (except for water) and smoking for at least 1 h before the tests.

### 3.2 Quantitative synthesis

In a qualitative analysis, all studies showed that elderly individuals required higher concentrations of solutions to perceive all flavors. Most studies indicated that the elderly individuals detected sweet taste as well as younger adults, suggesting that it might be a flavor that is easier to recognize; however, they still needed slightly higher concentrations to recognize it than younger adults.

Of the 6 studies included in the meta-analysis, 4 used the detection threshold method with the minimum concentration of the flavor perceived, where a lower value is better. The remaining 2 studies used the Likert scale with a numerical range, where the lowest value indicated no taste perception and the highest value indicated extreme taste perception, meaning that a higher value in this method was the positive outcome. For standardization purposes, the means of the studies using the Likert scale were converted to negative values since most of the studies included in the statistical analysis employed the method where a lower value was better for taste perception (30).

For standardization of the groups, we decided to maintain 2 groups (adults vs. elderly), as some studies included 3 groups with middle-aged adults, which were excluded from the analysis. Thus, there was one group of adults aged 18–40 years and one group of elderly individuals aged ≥ 60 years. Huang (2022) reported the mean (M) and standard deviation (SD) by sex in each group, and Weiffenbach (1986) provided the M and SD for elderly individuals in two different age groups. To standardize, the data was combined by sex in each group and the elderly groups were merged through the calculation of aggregated M and SD (30).

**Figure 2:**
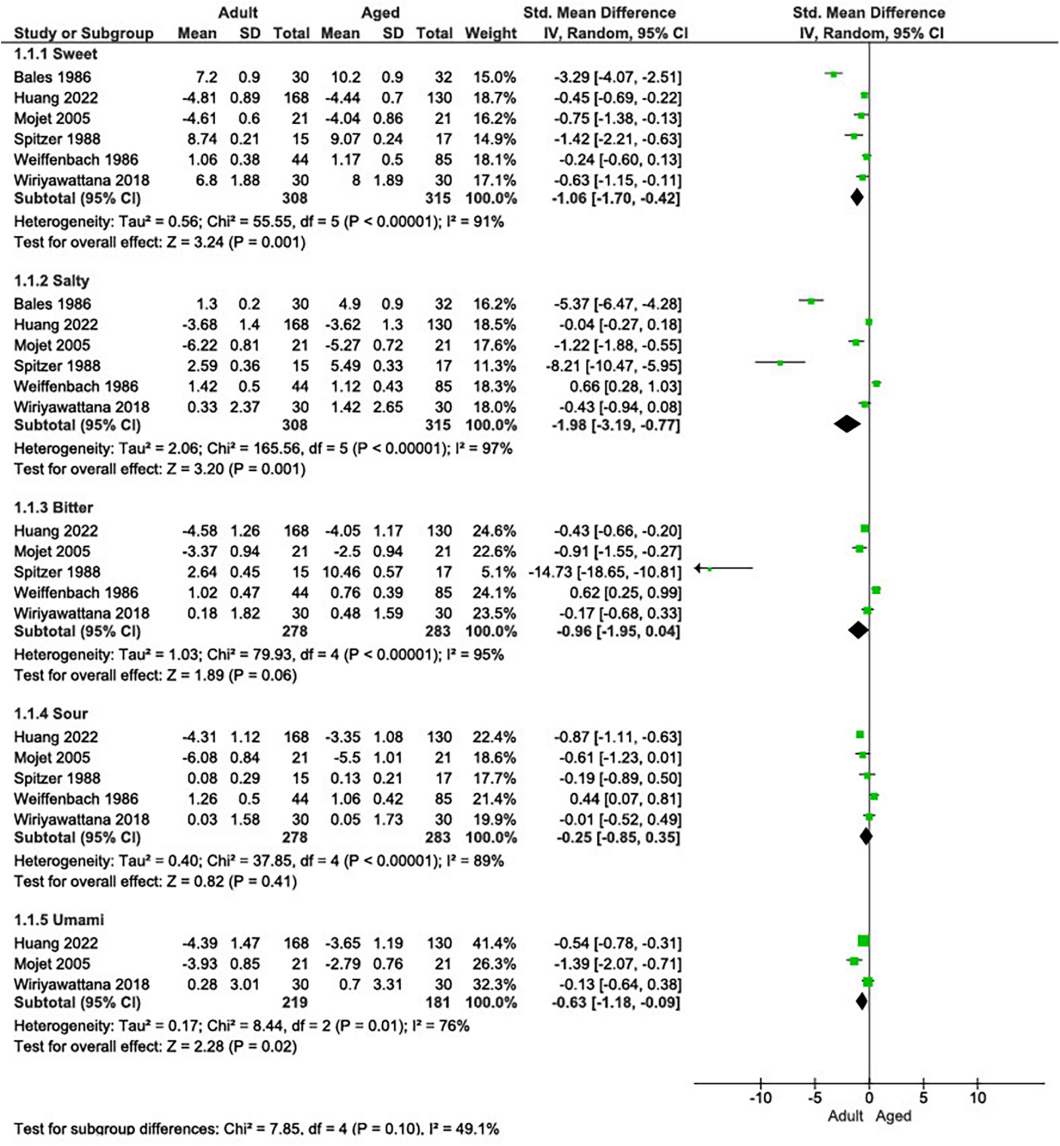
Forest plot of the five flavors perception (sweet, salty, bitter, sour, and umami) compared between adult and elderly groups.

The meta-analysis comparing the mean taste perception for each flavor between adults and elderly individuals is presented in Figure 2. There was a statistical difference for sweet, salty, and umami flavors, while sour and bitter flavors did not show a statistical difference between the groups. The sweet taste had a SMD of −1.06 [95% confidence interval (95% CI) −1.70, −0.42], salty taste had a SMD of −1.98 (95% CI −3.19, −0.77), bitter taste had a SMD of −0.96 (95% CI −1.95, 0.04), sour taste had a SMD of −0.25 (95%CI −0.85, 0.35), and umami taste a SMD of −0.63 (95% CI −1.18, −0.09).

The assessment of the risk of bias of the included studies is shown in Table 1. Details regarding the application of the instrument is shown in Supplementary Appendix 3. Applying the defined criteria resulted in 15 articles with low quality, and 4 with high quality. The item that most significantly impacted the evaluation of risk of bias were related to sample inclusion criteria, such as age, participants' health condition, and lifestyle.

## 4 Discussion

There are several studies in the literature that assess taste perception in relation to genes, certain nutritional deficiencies, age, among other comparisons. Here, we conducted a rigorous systematic review to examine whether elderly individuals exhibit changes in taste perception compared to adults. Our main result was that elderly individuals presented altered taste sensation, requiring higher concentrations to identify sweet (large effect size), salty (large effect size), and umami (moderate effect size) flavors compared to adults.

Although the meta-analysis showed a significant decrease in taste perception in elderly compared to adults, some studies reported that the recognition threshold for sweet taste was similar between the groups in the qualitative analysis. One hypothesis for this result is that there may be a higher consumption of sweet foods by elderly individuals. This increase in the consumption of sweeter foods could be explained by the need to perceive the taste, considering a loss of taste perception, or because sweet flavor is easier to recognize. However, when the averages were combined in the meta-analysis, only bitter and sour flavors did not show significant differences between the groups. Our results differ in two flavors from those reported in the systematic review by Methven et al. (2012) (12). In their meta-analysis, 20 out of the 23 included studies revealed higher taste thresholds for the elderly across all flavors, indicating a decline in taste perception with aging. A possible explanation for this discrepancy in results may be the number of studies included in each meta-analysis, as our eligibility criteria resulted in differences in the number of studies included. Specifically, we included three studies in our meta-analysis that were not part of theirs.

Murphy and Withee (1986) reported that elderly individuals rated sucrose and NaCl solutions at higher concentrations as more pleasant compared to younger individuals, due to this decrease in sensory perception (31). Our results confirm that the taste disorder may be related to the fact that elderly individuals reported finding high concentrations of sucrose and NaCl more pleasant. This could lead to an increase in the consumption of sugary and salty foods among the aged individuals. This potential impact their dietary choices can significantly impact individual's oral health outcomes, which should be better investigated. The increased consumption of sugary foods, in particular, can alter the oral microbiome, promoting the development of coronal and root caries and exacerbating periodontal diseases (32). In the case of dental caries, the microorganisms present benefit from the high frequency of sugar consumption, producing acid and resulting in an acidic pH that promotes the demineralization of the dental structure (33).

A hypothesis suggests that fermentable carbohydrates may also be a risk factor for periodontal diseases, with high consumption of added sugars linked to these conditions. This association persists even when considering other risk factors, indicating that sugar intake and postprandial hyperglycemia may contribute to systemic inflammation, impacting both periodontal diseases and other non-communicable diseases (32). Furthermore, it was recently reported that gingival solitary chemosensory cells (gSCCs) express bitter taste receptors (Tas2r) and are present also in the sulcular and junctional epithelia, where they can detect quorum-sensing acyl-homoserine lactone (AHL) molecules released from periodontal diseases-associated bacteria. Activation of Tas2r in gSCCs leads to the induction of antimicrobial proteins (AMPs). This activity is required for host-microbe homeostasis, since genetic ablation of a Tas2r-associated signaling component (α-gustducin; Gnat3) causes dysbiotic alterations to the commensal microbiota and naturally occurring periodontal bone loss (34).

Subsequently, the link between declining taste perception and its impact on oral and systemic health is evident. Caries and periodontitis increase the risk of tooth loss, which can significantly diminish an individual's quality of life, impair their ability to chew and digest food properly, and limit their nutritional intake. These changes in nutritional status and diet may further exacerbate systemic health conditions, such as cardiovascular disease, diabetes, and malnutrition, which, in turn, worsen oral health and perpetuate a harmful cycle. From a clinical perspective, this underscores the need for the dental team to monitor and manage declining taste perception and any related changes in sugar intake during aging to reduce the risk of dental diseases and support overall oral health. Incorporating a dietary analysis into the patient's record as a routine aspect of dental appointments can facilitate effective follow-up and promote healthy aging.

As previously stated, among the factors that can impair taste perception in elderly individuals are the use of medications and hyposalivation (35). The mechanisms responsible for the adverse effects of medication use on taste are not well understood; they are likely multiple and interactive. These effects can include changes in the taste of the medication itself, damage to taste receptors (such as from gastroesophageal reflux acid), influences from immunosuppression and related conditions like oral candidiasis, alterations in neurotransmitter function, drying of the oral mucosa, changes in the chemical composition of saliva and mucosal elements, among other adverse effects (36). Foods contain taste substances such as inorganic ions, polysaccharides, and amino acids (37). Some of these taste substances chemically interact with salivary components while accessing taste receptor sites. Additionally, certain salivary components continuously stimulate taste receptors and have a long-term effect, protecting the taste receptor site and/or taste buds (37). Taste sensitivity can be affected in various ways by saliva, including the stimulation of taste receptors, chemical interaction with taste substances, and protection of taste receptors (38). It was observed that individuals with taste disorders had significantly lower stimulated salivary flow, as measured by the gingival test, compared to individuals without taste disorders (39).

The limitations of this study include the use of various sensory methods, which reduces the ability to compile and compare results more robustly in a statistical analysis. Additionally, only cross-sectional studies were found. One study was deemed to be case-control; however, since the categorization was based on the independent variable (and not the outcome), we treated this study as cross-sectional. We could not find any longitudinal studies, which means that any inference of “cause-effect” is impossible at this moment. Furthermore, the included studies are geographically concentrated, which may limit the generalizability of findings across diverse elderly populations. As this review encompassed a thorough examination of the available literature, including multiple databases and grey literature, we understand that publication bias is unlikely, and that no cultural inferences or cause-effect relationships can be established at this point. However, the data found in the cross-sectional studies corroborate with the meta-analysis conducted, indicating a decline in taste perception in elderly individuals compared to adults and this can be related to oral health impairment. Future research could explore the development of long-term cohort studies that track changes in individuals' taste perception over time, along with any associated alterations in food preferences and oral health outcomes. Additionally, researchers should better explore patient-centered outcomes, such as taste perception, and examine their relationship with oral health, as our review found limited information on this topic.

In conclusion, there is a decline in taste perception in elderly individuals compared to adults for sweet, salty, and umami flavors. Bitter and sour flavors did not exhibit differences in elderly.

## Data Availability

The original contributions presented in the study are included in the article/Supplementary Material, further inquiries can be directed to the corresponding author.
