# Supplementary material for: Changes in taste perception in elderly population and its potential impact on oral health: a systematic review with meta-analysis
Source: Front Oral Health. 2024 Dec 5;5:1517913. doi: 10.3389/froh.2024.1517913 (PMC11655467; doi:10.3389/froh.2024.1517913)
Supplement: Supplementary file 1 [file Table1.docx]

**Changes in taste perception in elderly population and its potential impact on oral health:**

**A systematic review with meta-analysis**

**Appendix 1.** Search strategies according to each database, performed on 16^th^ July 2023

| **Pubmed** | ((("Old Age"[All Fields] OR "Old Aged"[All Fields] OR "Old Elder"[All Fields] OR "Old Elderly"[All Fields] OR "Ageing"[All Fields] OR "Older adults"[All Fields] OR "old adults"[All Fields] OR "Aged"[MeSH Terms]) AND ("Taste Disorders"[All Fields] OR "Dysgeusia"[All Fields] OR "Hedonic Scale"[All Fields] OR "Tasting"[All Fields] OR "Dysgeusias"[All Fields] OR "Distorted Taste"[All Fields] OR "Altered Taste"[All Fields] OR "Parageusia"[All Fields] OR "Parageusias"[All Fields] OR "taste sensation"[All Fields] OR "Taste Perception"[All Fields] OR "Gustatory Perception"[All Fields] OR "Gustatory Response"[All Fields] OR "sweet taste"[All Fields] OR "diet, carbohydrate restricted"[MeSH Terms] OR "Dietary Sugars"[MeSH Terms])) NOT "covid 19"[MeSH Terms]) NOT ("Review"[Publication Type] OR "systematic  review"[Publication Type]) | 2218 |
| --- | --- | --- |
| **Scopus** | ( ABS ( "Old Age" OR "Old Aged" OR "Old Elder" OR "Old Elderly" OR "Ageing" OR "Older adults" OR "old adults" OR "Aged" ) AND ABS ( "Taste Disorders" OR dysgeusia OR "Hedonic Scale" OR tasting OR "Dysgeusias" OR "Distorted Taste" OR "Altered Taste" OR parageusia OR parageusias OR "Taste sensation" OR "Taste Perception" OR "Gustatory Perception" OR "Gustatory Response" OR "diet, carbohydrate restricted" OR "sweet taste" OR "Dietary Sugars") ) | 810 |
| **ProQuest** | noft(((("Old Age" OR "Old Aged" OR "Old Elder" OR "Old Elderly" OR "Ageing" OR "Older adults" OR "old adults" OR "Aged") AND ("Taste Disorders" OR "Dysgeusia" OR "Hedonic Scale" OR "Tasting" OR "Dysgeusias" OR "Distorted Taste" OR "Altered Taste" OR "Parageusia" OR "Parageusias" OR "taste sensation" OR "Taste Perception" OR "Gustatory Perception" OR "Gustatory Response" OR "sweet taste" OR "diet, carbohydrate restricted" OR "Dietary Sugars")) NOT ("covid 19") NOT ("Review" OR "systematic review"))) | 163 |
| **Web of Science** | "Old Age" OR "Old Aged" OR "Old Elder" OR "Old Elderly" OR "Ageing" OR "Older adults" OR "old adults" OR "Aged" (Abstract) and "Taste Disorders" OR "Dysgeusia" OR "Hedonic Scale" OR "Tasting" OR "dysgeusia" OR "Distorted Taste" OR "Altered Taste" OR "parageusie" OR "parageusie" OR "taste sensation" OR "Taste Perception" OR "Gustatory Perception" OR "Gustatory Response" OR "sweet taste" OR "diet,  carbohydrate restricted" OR "Dietary Sugars" (Title) | 1367 |
| **Embase** | ('old age':ti,ab,kw OR 'old aged':ti,ab,kw OR 'old elder':ti,ab,kw OR 'old elderly':ti,ab,kw OR 'ageing':ti,ab,kw OR 'older adults':ti,ab,kw OR 'old adults':ti,ab,kw OR 'aged':ti,ab,kw) AND ('taste disorders':ti,ab,kw OR 'dysgeusia':ti,ab,kw OR 'hedonic scale':ti,ab,kw OR 'tasting':ti,ab,kw OR 'dysgeusias':ti,ab,kw OR 'distorted taste':ti,ab,kw OR 'altered taste':ti,ab,kw OR 'parageusia':ti,ab,kw OR 'parageusias':ti,ab,kw OR 'taste sensation':ti,ab,kw OR 'taste perception':ti,ab,kw OR 'gustatory perception':ti,ab,kw OR  'gustatory response':ti,ab,kw OR 'sweet taste':ti,ab,kw) NOT 'covid 19':ti,ab,kw NOT ('review':ti,ab,kw OR 'systematic review':ti,ab,kw) | 630 |
| **LIVIVO** | ((("Old Age" OR "Old Aged" OR "Old Elder" OR "Old Elderly" OR "Ageing" OR "Older adults" OR "old adults" OR "Aged”) AND ("Taste Disorders" OR "Dysgeusia" OR "Hedonic Scale" OR "Tasting" OR "Dysgeusias" OR "Distorted Taste" OR "Altered Taste" OR "Parageusia" OR "Parageusias" OR "taste sensation" OR "Taste Perception" OR "Gustatory Perception" OR "Gustatory Response" OR "sweet taste" OR "diet,  carbohydrate restricted" OR "Dietary Sugars")) NOT ("covid 19") NOT ("Review" OR "systematic review")) | 372 |

| **Google Scholar** | ((“Old Age” OR “Older adults”) AND (“Taste Disorders” OR “Altered Taste” OR "diet, carbohydrate restricted")) -"Covid-19" | First 100 out of 2,670. |
| --- | --- | --- |

**Appendix 2.** Excluded articles in phase 2 and reasons for exclusion (n= 46)

| **Author, year** | **Reason for exclusion** |
| --- | --- |
| *After full-text reading* |  |
| (1) (Alia et al. 2021) | 3 |
| (2) (Allen et al. 2011) | 2 |
| (3) (Appleton et al. 2016) | 1 |
| (4) (Bartoshuk et al. 1986) | 1 |
| (5) (Boesveldt et al. 2011) | 3 |
| (6) (Cao et al. 2015) | 2 |
| (7) (Cohen et al. 1959) | 1 |
| (8) (Coltell et al. 2019) | 3 |
| (9) (Dias et al. 2015) | 3 |
| (10) (Dikmen et al. 2017) | 2 |
| (11) (Drewnowski et al. 2001) | 2 |
| (12) ( Fikentscher et al. 1977) | 3 |
| (13) (Gervis et al. 2020) | 2 |
| (14) (Gervis et al. 2021) | 3 |
| (15) (Guido et al. 2016 ) | 3 |
| (16) (Habberstad et al. 2017) | 3 |
| (17) (Hasan et al. 2022) | 3 |
| (18) ( Hyde R. J., Feller R. P. 1981) | 2 |
| (19) ( Jacquot et al. 2020) | 1 |
| (20) (Jayasinghe et al. 2017) | 3 |
| (21) (Jayasinghe et al. 2017) | 2 |
| (22) (Kanjirath et al. 2018) | 2 |
| (23) (Klimacka-Nawrot et al. 2005) | 2 |

| (24) | (Lampuré et al. 2015) | 3 |
| --- | --- | --- |
| (25) | (Magdalena et al. 2017) | 2 |
| (26) | (Martelli et al. 2020) | 3 |
| (27) | (Mattos et al. 2022) | 3 |
| (28) | (Methven et al. 2012) | 2 |
| (29) | (Miotti et al. 1989) | 2 |
| (30) | (Mondon et al. 2014) | 2 |
| (31) | (Fatemeh et al. 2021) | 3 |
| (32) | (Nagy et al. 2014) | 3 |
| (33) | (Ogawa et al. 2016) | 3 |
| (34) | (Ogawa et al. 2017) | 3 |
| (35) | (Ozturk et al. 2022) | 3 |
| (36) | (Pfrimer et al. 2023) | 2 |
| (37) | (Plattig et al. 1980) | 2 |
| (38) | (Richter et al. 2017) | 2 |
| (39) | (Rivers et al. 2017) | 2 |
| (40) | (Robino et al. 2015) | 3 |
| (41) | (Sergi et al. 2017) | 2 |
| (42) | (Silva et al. 2021) | 3 |
| (43) | (Stevens et al. 1993) | 4 |
| (44) | (Trachootham et al. 2018) | 3 |
| (45) | (Uota et al. 2016) | 3 |
| (46) | (Yoshinaka et al. 2016) | 1 |

1- Studies including children and adolescents, edentulous individuals, participants with cancer or those who have undergone chemotherapy and radiation therapy, individuals with dementia or other neurological conditions, and participants who are continuously using medications.

2 = Articles with full text not available, Reviews, Review systematic, Book chapters, opinions, letters, conference abstracts, study protocols, case reports, case series, duplicate data.

3 = Studies without a control group (adults) or test group (elderly).

4 = Studies that evaluated did not evaluate the sweet taste.

5 = Studies that have not assessed taste perception using a quantitative/qualitative scale.

# Excluded articles references

1. Alia S, Aquilanti L, Pugnaloni S, Di Paolo A, Rappelli G, Vignini A. The influence of age and oral health on taste perception in older adults: A case-control study. Nutrients. 2021;13(11):1–9.
2. Allen VJ, Withers C, Gosney MA, Methven L. Assessment of the liking of food products in two elderly cohorts. Age Ageing [Internet]. 2011;40:i72.
3. Appleton KM, Smith E. A Role for Identification in the Gradual Decline in the Pleasantness of Flavors with Age. Journals Gerontol - Ser B Psychol Sci Soc Sci. 2016;71(6):987–94.
4. Bartoshuk LM, Rifkin B, Marks LE, Bars P. Taste and aging. Journals Gerontol. 1986;41(1):51–7.
5. Boesveldt S, Lindau ST, McClintock M k., Hummel T, Lundström JN. Probability Study. Natl Geogr Mag. 2013;49(3):324–30.
6. Cao L, Kawai M, Uneyama H. Development of an easy taste test using whole mouth tasting procedure. Chem Senses [Internet]. 2015;40(8):590.
7. Cohen T, Gitman L. Oral complaints and taste perception in the aged. J Gerontol. 1959;14(9):294–8.
8. Coltell O, Sorlí J V., Asensio EM, Fernández-Carrión R, Barragán R, Ortega-Azorín C, et al. Association between taste perception and adiposity in overweight or obese older subjects with metabolic syndrome and identification of novel taste-related genes. Am J Clin Nutr [Internet]. 2019;109(6):1709–23.
9. Dias AG, Eny KM, Cockburn M, Chiu W, Nielsen DE, Duizer L, et al. Variation in the TAS1R2 Gene, Sweet Taste Perception and Intake of Sugars. J Nutrigenet Nutrigenomics. 2015;8(2):81–90.
10. Dikmen D, Inan-Eroglu E, Ozdemir A, Madali B, Ellahi B. The impact of taste preferences on macronutrient intake. Ann Nutr Metab [Internet]. 2017;71:1134–5.
11. Drewnowski A, Shultz JM. Impact of aging on eating behaviors, food choices, nutrition, and health status. J Nutr Heal Aging [Internet]. 2001;5(2):75–9.
12. FIKENTSCHER R, ROSEBURG B, SPINAR H, BRUCHMÜLLER W. Loss of taste in the elderly: sex differences. Clin Otolaryngol Allied Sci. 1977;2(3):183–9.
13. Gervis JE, Chui KKH, Coltell O, Fernández-Carrión R, Barragán R, Corella D, et al. Taste perception profiles are associated with adiposity among older adults with metabolic syndrome-PREDIMED- Plus. Chem Senses [Internet]. 2020;45:712–3.
14. Gervis JE, Chui KKH, Ma J, Coltell O, Fernández-Carrión R, Sorlí J V., et al. Data-Driven Clustering Approach to Derive Taste Perception Profiles from Sweet, Salt, Sour, Bitter, and Umami Perception Scores: An Illustration among Older Adults with Metabolic Syndrome. J Nutr. 2021;151(9):2843–51.
15. Guido D, Perna S, Carrai M, Barale R, Grassi M, Rondanelli M. Multidimensional evaluation of endogenous and health factors affecting food preferences, taste and smell perception. J Nutr Heal Aging. 2016;20(10):971–81.
16. Habberstad C, Drake I, Sonestedt E. Variation in the sweet taste receptor gene and dietary intake in a Swedish middle-aged population. Front Endocrinol (Lausanne). 2017;8(DEC).
17. Hasan A, L P, Kalra P, M A. A study of taste alterations in type 2 diabetes mellitus patients with a good glycemic control. Natl J Physiol Pharm Pharmacol. 2022;12(0):1.
18. Hyde RJ, Feller RP. Age and sex effects on taste of sucrose, NaCl, citric acid and caffeine. Neurobiol Aging. 1981;2(4):315–8.
19. Jacquot L, Bereau M. Vieillissement et troubles du goût et de l’olfaction. Soins Gérontologie

[Internet]. setembro de 2020;6851(145):1 YP – 48.

1. Jayasinghe SN, Kruger R, Walsh DCI, Rivers S, Breier BH. Does sweet taste perception explain habitual sweet food liking and choice? Nutrients [Internet]. 2017;9(3).
2. Jayasinghe SN, Kruger R, Walsh DCI, Cao G, Rivers S, Richter M, et al. Is sweet taste perception associated with sweet food liking and intake? Nutrients. 2017;9(7):1–19.
3. Kanjirath P, Williams L. The impact of taste perception on the health of the aging population. Gen Dent. 2018;66(4):24–5.
4. Klimacka-Nawrot E, Suchecka W, Błońska-Fajfrowska B. Relationship between sweetness liking, taste sensitivity and sweetening habits in adults. Wiad Lek [Internet]. 2005;58(3–4):174–9.
5. Lampuré A, Schlich P, Deglaire A, Castetbon K, Péneau S, Hercberg S, et al. Sociodemographic, psychological, and lifestyle characteristics are associated with a liking for salty and sweet tastes in French adults. J Nutr. 2015;145(3):587–94.
6. Magdalena S a, Aleksandra S, Monika SK, Ma_gorzata K mierz;, Stanis_aw S a; M ca, Hanna K. Taste disorders in the elderly. J Educ Heal Sport. 2017;7(0):01–01.
7. Martelli ME, Jacob N, Morais MA, da-Cunha DT, Corona LP, Capitani CD, et al. Taste sensitivity throughout age and the relationship with the sleep quality. Sleep Sci. 2020;13(1):32–6.
8. Mattos JL, Hasan S, Schlosser RJ, Payne SC, Soler ZM. The association of gustatory dysfunction, olfactory dysfunction, and cognition in older adults. Int Forum Allergy Rhinol. 2023;13(9):1577– 83.
9. Methven L, Allen VJ, Withers CA, Gosney MA. Ageing and taste. Proc Nutr Soc. 2012;71(4):556– 65.
10. Miotti A, Passi P, Carli PO, Giannuzzi Savelli L, Isola A. Taste changes in diabetics. Clinico- experimental study. G Stomatol Ortognatodonzia. 1988;8(4):41–9.
11. Mondon K, Naudin M, Beaufils É, Atanasova B. Perception of taste and smell in normal and pathological aging: An update. Geriatr Psychol Neuropsychiatr Vieil. 2014;12(3):313–20.
12. Mozhdehi FJ, Abeywickrema S, Bremer PJ, Peng M. Following Vegan , Vegetarian , or Omnivore Diets. 2021;
13. Nagy A, Steele CM, Pelletier CA. Differences in swallowing between high and low concentration taste stimuli. Biomed Res Int. 2014;2014.
14. Ogawa T, Uota M, Ikebe K, Notomi Y, Iwamoto Y, Shirobayashi I, et al. Taste detection ability of elderly nursing home residents. J Oral Rehabil. 2016;43(7):505–10.
15. Ogawa T, Uota M, Ikebe K, Arai Y, Kamide K, Gondo Y, et al. Longitudinal study of factors affecting taste sense decline in old-old individuals. J Oral Rehabil. 2017;44(1):22–9.
16. Ozturk EE, Ozturk ZA. The Influence of Nutritional Status and Sleep Quality on Gustatory Function in Older Adults. Med. 2023;59(1):1–11.
17. Pfrimer K, dos Santos GR, Costa TMB, Lucca APB. Perception of sweet taste in people with type 2 diabetics. Clin Nutr ESPEN [Internet]. 2023;54:707–8.
18. Plattig KH, Kobal G, Thumfart W. The chemical senses of smell and taste in the course of life - changes of smell and taste perception. Z Gerontol. 1980;13(2):149–57.
19. Richter M, Cao G, Jayasinghe S, Kruger R, Bernhard B, Walsh D. Do measures of sweet taste intensity and hedonic liking of glucose play a role in dietary intake of sweet food? Ann Nutr Metab [Internet]. 2017;71:288–9.
20. Rivers S, Jayasinghe SN, Kruger R, Breier BH. Habitual sweet food and beverage intake is influenced by perception of sweet taste intensity. Nutrients [Internet]. 2017;9(3).
21. Robino A, Bevilacqua L, Pirastu N, Situlin R, Di R, Paolo L, et al. Polymorphisms in sweet taste genes ( TAS1R2 and GLUT2 ), sweet liking , and dental caries prevalence in an adult Italian population. Genes Nutr. 2015;10(5):1–9.
22. Sergi G, Bano G, Pizzato S, Veronese N, Manzato E. Taste loss in the elderly: Possible implications for dietary habits. Crit Rev Food Sci Nutr. 2017;57(17):3684–9.
23. Silva ROC, Lacerda WF, Henn IW, Chaiben CL, Machado MÂN, de Lima AAS. Relationship between taste perception and use of upper complete dentures. Spec Care Dent. 2021;41(2):244–50.
24. Stevens JC, Cain WS. Changes in Taste and Flavor in Aging. Crit Rev Food Sci Nutr. 1993;33(1):27–37.
25. Trachootham D, Satoh-Kuriwada S, Lam-ubol A, Promkam C, Chotechuang N, Sasano T, et al. Differences in taste perception and spicy preference: A thai-japanese cross-cultural study. Chem Senses. 2018;43(1):65–74.
26. Uota M, Ogawa T, Ikebe K, Arai Y, Kamide K, Gondo Y, et al. Factors related to taste sensitivity in elderly: cross-sectional findings from SONIC study. J Oral Rehabil. 2016;43(12):943–52.
27. Yoshinaka M, Ikebe K, Uota M, Ogawa T, Okada T, Inomata C, et al. Age and sex differences in the taste sensitivity of young adult, young-old and old-old Japanese. Geriatr Gerontol Int. 2016;16(12):1281–8.

**Appendix 3.** Risk of bias assessment of individual studies using the JBI Critical Appraisal Checklist for Analytical Cross-Sectional Studies (Moola et al. 2020).

| Author | 1. Were the criteria for inclusion in the sample clearly defined? | 2. Were the study subjects and the setting described in detail? | 3. Was the exposure measured in a valid and reliable way? | 4. Were objective, standard criteria used for measurement of the condition? | 5. Were confounding factors identified? | 6. Were strategies to deal with confounding factors stated? | 7. Were the outcomes measured in a valid and reliable way? | 8. Was appropriate statistical analysis used? | **Overall appraisal:**  LOW, MODERATE, OR HIGH RISK |
| --- | --- | --- | --- | --- | --- | --- | --- | --- | --- |
| **Barragán, 2018** | U | U | Y | Y | N | N | Y | Y | **HIGH RISK** |
| **Bales, 1986** | U | U | Y | Y | N | N | U | Y | **HIGH RISK** |
| **Cowart, 1989** | U | U | Y | U | N | N | U | Y | **HIGH RISK** |
| **Fukunaga, 2005** | U | U | Y | Y | N | N | U | Y | **HIGH RISK** |
| **Gilmore, 1989** | U | U | U | Y | N | N | Y | Y | **HIGH RISK** |
| **Huang, 2022** | Y | Y | Y | Y | Y | Y | Y | Y | **LOW RISK** |
| **Jeon, 2021** | U | Y | Y | U | U | N | Y | Y | **HIGH RISK** |
| **Keneda, 2000** | N | U | U | U | N | N | N | N | **HIGH RISK** |
| **Kennedy, 2010** | N | U | Y | N | N | N | U | Y | **HIGH RISK** |
| **Mojet, 2001** | Y | U | Y | Y | Y | Y | Y | Y | **LOW RISK** |
| **Mojet, 2003** | Y | U | Y | Y | Y | Y | Y | Y | **LOW RISK** |
| **Mojet, 2005** | Y | U | Y | Y | Y | Y | Y | Y | **LOW RISK** |
| **Receputo, 1996** | N | N | U | N | N | N | U | N | **HIGH RISK** |
| **Spitz, 1988** | N | U | Y | Y | N | N | Y | Y | **HIGH RISK** |
| **Stevens, 1995** | N | U | Y | U | N | N | Y | Y | **HIGH RISK** |
| **Wang, 2020** | Y | N | Y | Y | N | N | Y | Y | **HIGH RISK** |
| **Weiffenbach, 1986** | N | U | Y | U | N | N | U | Y | **HIGH RISK** |
| **Wiriyawattana, 2018** | U | U | Y | Y | Y | N | Y | Y | **HIGH RISK** |

Y: Yes; N: No; U: Unclear; NA: Not applicable.

1. 1. Were the criteria for inclusion in the sample clearly defined? **CRITICAL DOMAIN**
2. 2. Were the study subjects and the setting described in detail? **VERY CRITICAL DOMAIN**
3. 3. Was the exposure measured in a valid and reliable way? **CRITICAL DOMAIN**
4. 4. Were objective, standard criteria used for measurement of the condition? **VERY CRITICAL DOMAIN**
5. 5. Were confounding factors identified? **NON-CRITICAL DOMAIN**
6. 6. Were strategies to deal with confounding factors stated? **NON-CRITICAL DOMAIN**
7. 7. Were the outcomes measured in a valid and reliable way? **NON-CRITICAL DOMAIN**
8. 8. Was appropriate statistical analysis used? **NON-CRITICAL DOMAIN**
